# Genotype–phenotype correlation in a large cohort of pediatric patients with heterozygous and homozygous familial hypercholesterolemia

**DOI:** 10.1097/MOL.0000000000000863

**Published:** 2023-04-01

**Authors:** M.D. Reijman, J.C. Defesche, A. Wiegman

**Affiliations:** a Department of Pediatrics; b Department of Human Genetics, Amsterdam UMC, University of Amsterdam, Amsterdam, The Netherlands

**Keywords:** cardiovascular risk, familial hypercholesterolemia, genetic diagnosis, heterozygous, homozygous, low-density lipoprotein cholesterol, pediatrics

## Abstract

**Background:**

Familial hypercholesterolemia (FH) is a genetic disorder characterized by elevated low-density lipoprotein cholesterol (LDL-C) levels and premature cardiovascular disease (CVD). Both the heterozygous form and the very severe homozygous form can be diagnosed by genetic testing and by clinical criteria. Genetic testing can discern FH in a form caused by complete absence of the LDL-receptors, the negative variant and a form leading to reduced activity of the LDL receptors, the defective variant. The aim of this study is to provide more insight in the genotype–phenotype correlation in children and adolescents diagnosed with heterozygous FH (HeFH) and with homozygous FH (HoFH), specifically in relation to the clinical and therapeutic consequences of the negative and defective variant of FH.

**Methods and Results:**

Data of 5904 children with a tentative diagnosis of FH referred to our center for genetic testing were collected. A lipid-profile was present in 3494 children, who became the study cohort. In this large cohort of children, which includes 2714 HeFH and 41 HoFH patients, it is shown that receptor negative variants are associated with significant higher LDL-C levels in HeFH patients than receptor defective variants (6.0 versus 4.9 mmol/L; *p* < 0.001). A negative/negative variant is associated with a significant higher LDL-C level jn HoFH patients than a negative/defective variant, which in itself has a higher LDL-C level than a defective/defective variant. Significantly more premature CVD is present in close relatives of children with HeFH with negative variants compared to close relatives of HeFH children with defective variants (75% vs 59%; *p *< 0.001).

**Conclusions:**

Performing genetic testing and identifying the type of underlying genetic variant is of added value in order to distinguish between pediatric patients with higher risks of premature CVD and to identify those that will benefit most from new types of lipid-lowering therapies. Since in children the phenotype of FH is less affected by environmental factors, the study substantiates the genotype-phenotype correlation in this large pediatric population.

## INTRODUCTION

Familial hypercholesterolemia (FH) is the most frequent autosomal dominant metabolic disorder in the world, affecting lipoprotein metabolism and is characterized by elevated low-density lipoprotein cholesterol (LDL-C) levels [1]. The most common form is heterozygous FH (HeFH) with a prevalence of approximately one in 300 births [2,3], characterized by elevated LDL-C of 2–3 times the level of normal and leading to cardiovascular disease (CVD) already from young adulthood onwards [4,5]. The second form is homozygous FH (HoFH), a rare condition occurring in about one in 300 000 births [2,6], associated with severely elevated LDL-C of 4–10 times the level of normal that can lead to CVD already in childhood [7].

HeFH and HoFH can be diagnosed both genetically and clinically. Genetically, HeFH is confirmed when a single pathogenic variant is found on the *LDLR*, *APOB* or *PCSK9* gene. When two pathogenic variants are present on different alleles of *LDLR*, *APOB*, or *PCSK9* genes, the diagnosis of HoFH can be made. When two pathogenic variants are present on both of the *LDLRAP1* genes, the very rare autosomal recessive hypercholesterolemia (ARH) is diagnosed [7]. A clinical diagnosis is based on one or several of the following conditions: elevated LDL-C levels, CVD, and clinical signs of FH (xanthoma or corneal arcus) in the patient or in the family of the patient [8].

HoFH is most often specified as true homozygous (two identical pathogenic variants on both alleles), compound heterozygous (two different pathogenic variants on both alleles of the same gene), and double heterozygous (two pathogenic variants in different genes) [8]. An alternative way to classify HoFH, and HeFH as well, is to specify categories based on the residual activity. Either the pathogenic variant results in failure to synthesize functional protein or in synthesis of a completely inactive protein, called a negative variant. Or the pathogenic variant results in a partially inactive protein, called a defective variant [9]. Negative variants cause overall higher LDL-C levels compared to defective variants in children with HeFH [10], and the more negative variants in HoFH patients exist, the higher LDL-C levels expected.

In our country, genetic testing for FH is the preferred method to confirm or to rule out FH. Genetic testing is also used for cascade screening in our country. This has resulted in the largest cohort of genetically diagnosed pediatric FH patients described from a single center. We earlier published data of our growing cohort, in 2003 in 1034 children [11], in 2011 in 1430 children [12], and to date the cohort has expanded to 5904 children. Studying data in a large cohort of children provides more accurate information about the genotype**–**phenotype correlation in FH, because LDL-C levels are less affected by environmental factors compared to adult patients [10]. The aim of this study is to assess the genotype**–**phenotype correlation in children and adolescents diagnosed with HeFH and HoFH and the clinical implications with regard to CVD risk, therapeutic consequences and long-term prospects. 

**Box 1:**
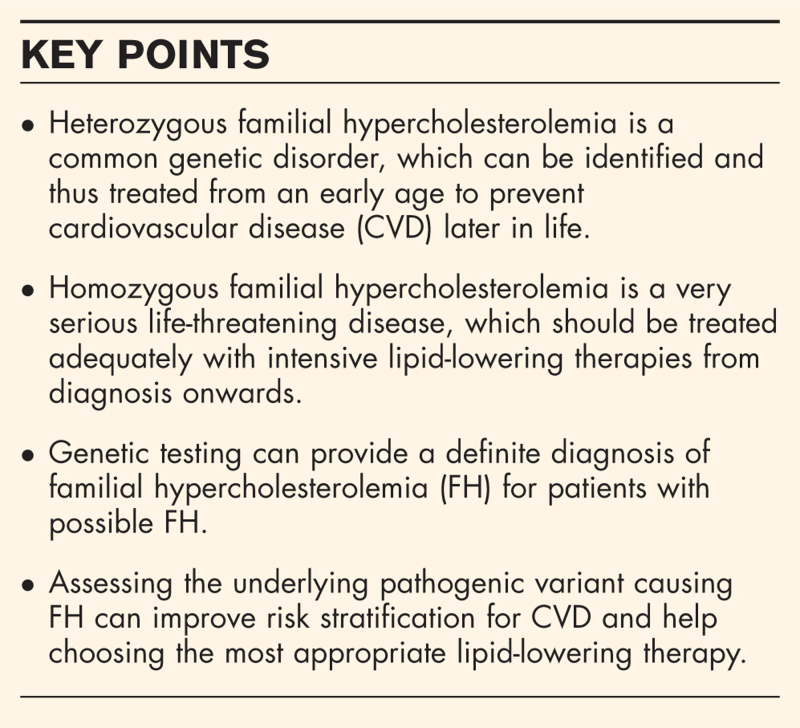
no caption available

## METHODS

### Study population

For this study data collected between July 1989 and January 2022 of patients 0–19 years of age with a tentative diagnosis of FH were used. These children and adolescents visited our pediatric lipid clinic or were referred by pediatricians and general practitioners as index patients for genetic testing for FH. The study population consisted of patients with genetically confirmed HeFH and HoFH, genetically confirmed non-FH (non-affected siblings) and patients clinically diagnosed with HeFH without a known pathogenic variant. Clinical diagnosis was made based on the criteria described by Wiegman *et al.*[13]. For comparison reasons, baseline cholesterol levels were mandatory, so patients without untreated LDL-C nor untreated total cholesterol were excluded. Genetically diagnosed patients with a genetic variant that turned out to be nonpathogenic were excluded, as well.

### Baseline characteristics/data collection

Demographic and clinical characteristics were collected during the first visit at the outpatient clinic, or data were collected from request forms for genetic testing. Cholesterol levels were measured as mmol/L.

### Type of underlying pathogenic variant

All pathogenic variants were rated as receptor negative or receptor defective. Pathogenic variants in the *LDLR* gene with no residual LDLR activity (<2%) were rated as negative variants. These included variants that resulted in the complete abolishment of protein synthesis, intracellular transport, ligand binding or receptor clustering. Variants in the *LDLR* gene rated as defective variants had residual LDLR activity (2–70%). These included reduced intracellular transport, reduced clustering or reduced recycling [14]. Variants in the *ApoB* gene (binding defect) and variants in the *PCSK9* gene (recycling defect) were rated as defective, according to their residual activity.

### Statistical analysis

Statistical analysis was performed using R version 4.0.3. Untreated mean LDL-C levels were compared between groups based on diagnosis; genetically confirmed HeFH and HoFH, genetically confirmed non-FH, and clinical HeFH without a known pathogenic variant. For those with genetically confirmed diagnoses, the groups were further specified based on the type of pathogenic variant (defective/negative). Furthermore, cardiovascular disease in relatives was compared between HeFH patients with a negative variant and HeFH patients with a defective variant. For this comparison, data of one patient per family were used. The Student's *t*-test was used to compare means between two groups and the one way ANOVA was used to compare means among three or more. For categorical variables, the chi-square test was used.

## RESULTS

### Study population

**FIGURE 1:**
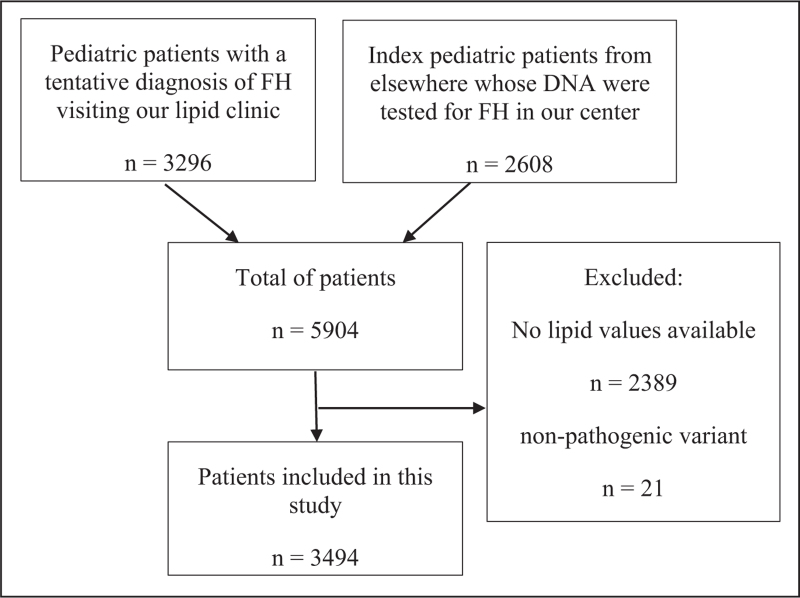
Flowchart of inclusion of the pediatric study population.

In total, data of 5904 subjects were collected (Fig. 1). Subjects with missing lipid values and those with nonpathogenic variants were excluded, after which 3494 subjects were included in the study.

### Baseline characteristics

Description of the baseline characteristics of the study population specified per patient group is presented in Table 1. Of the 3494 subjects, 2714 had genetically confirmed HeFH: 90.7% in *LDLR* gene, 9.0% in *APOB* gene and 0.3% in *PCSK9* gene; 41 patients had genetically confirmed HoFH; 583 were non-affected siblings (genetically confirmed non-FH); and 156 had a clinical diagnosis of HeFH but without a known pathogenic variant yet found. The percentage of male patients varied between 41 and 50%. The median age at diagnosis varied between 9.5 and 12.1 years of age and was the lowest in the group of HoFH patients. Mean (SD) untreated LDL-C levels were 5.4 (1.5), 12.1 (5.4), 2.5 (0.6), and 4.7 (0.9) mmol/l for genetically confirmed HeFH, and HoFH, genetically confirmed non-FH, and clinically diagnosed HeFH, respectively. In the genetically confirmed HeFH patients, a total of 275 different types of pathogenic variants were detected from 1748 apparently unrelated families (Supplementary Table 1). Stigmata were seen in 43% of the children with HoFH, in 2.6% of the children with genetically confirmed and in 3.1% of the children with clinically diagnosed HeFH.

**Table 1:** Baseline characteristics by diagnosis

	Genetically confirmed heterozygous FH	Clinically diagnosed heterozygous FH without a known pathogenic variant	p-value	Genetically confirmed non-FH	Genetically confirmed homozygous FH
N	2714	156		583	41^a^
Male - n (%)	1359 (50.1)	64 (41.0)	0.191	289 (49.6)	17 (41.5)
Age (median [IQR])	10.3 [7.7, 13.4]	12.1 [9.1, 15.4]	<0.001	10.3 [7.3, 13.3]	9.5 [4.9, 13.2]
Type of variant (%)					
Defective	1512 (55.7)				
Negative	1202 (44.3)				
Defective/defective					14 (34.1)
Negative/defective					15 (36.6)
Negative/negative					12 (29.3)
Smoking - n (%)	69 / 2525 (2.7)	3 / 124 (2.4)	0.404	14 / 521 (2.7)	1 / 33 (3.0)
BMI^b^ (median [IQR])	17.2 [15.7, 19.8]	21.3 [18.6, 24.9]	<0.001	16.9 [15.5, 19.5]	16.6 [15.5, 20.1]
Diabetes mellitus - n (%)	10 / 2493 (0.4)	3 / 123 (2.4)	<0.001	0 / 449 (0)	1 / 33 (3.0)
Stigmata - n (%)	66 / 2539 (2.6)	4 / 131 (3.1)	0.414	0 / 544 (0)	16 / 37 (43.2)
TC (mean (SD)) – mmol/L	7.1 (1.5)	6.6 (1.1)	<0.001	4.2 (0.7)	13.6 (5.3)
LDL-C (mean (SD)) – mmol/l	5.4 (1.5)	4.7 (0.9)	<0.001	2.5 (0.6)	12.1 (5.4)
HDL-C (mean (SD) – mmol/L	1.4 (0.3)	1.3 (0.3)	0.474	1.4 (0.3)	1.1 (0.4)
TG (median [IQR]) – mmol/L	0.7 [0.5, 1.0]	1.1 [0.7, 1.6]	<0.001	0.6 [0.4, 0.9]	0.9 [0.6, 1.4]

a Two ARH, autosomal recessive hypercholesterolemia.

b Median BMI increases with age, p corrected for age. BMI, body mass index; FH, familial hypercholesterolaemia; HDL-C, high-density lipoprotein cholesterol; HeFH, heterozygous familial hypercholesterolaemia; IQR, interquartile range; LDL-C, low-density lipoprotein cholesterol; TC, total cholesterol; TG, triglycerides.

### Low-density lipoprotein cholesterol by type of underlying pathogenic variant

**FIGURE 2:**
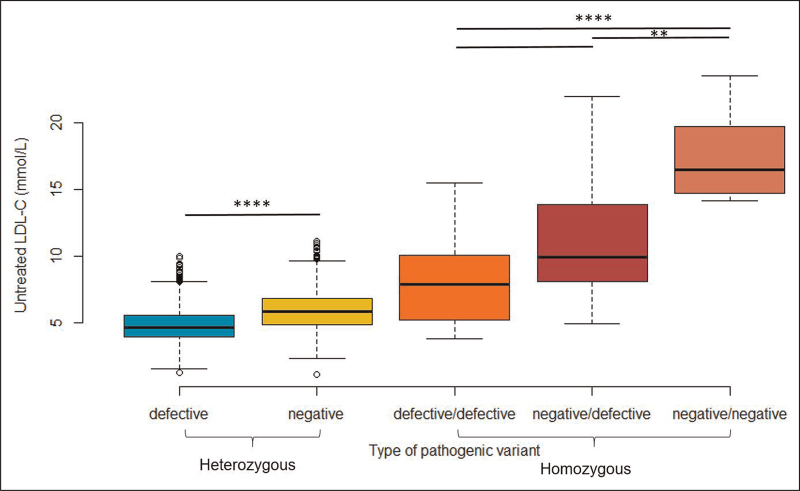
mean LDL-C by type of pathogenic variant for HeFH and HoFH.

Of the 2714 genetically confirmed HeFH patients, 1202 had an underlying pathogenic variant rated as negative and 1512 patients had a defective variant (Supplementary Table 1). As shown in Fig. 2 and Table 2, patients with a negative variant had a significantly higher mean (SD) LDL-C level at diagnosis of 6.0 (1.4) mmol/l compared to 4.9 (1.3) mmol/l for patients with a defective variant (*P* < 0.001). Clinically diagnosed HeFH patients without a known pathogenic variant had even mean LDL-C levels of 4.7 (0.9) mmol/l, even significantly lower than a defective variant (*p* < 0.001).

**Table 2:** mean LDL-C by type of pathogenic variant for HeFH and HoFH

	Count	Mean LDL-C	SD
Defective - mmol/L	1512	4.9	1.3
Negative - mmol/L	1202	6.0	1.4
Defective/defective - mmol/L	14	8.4	3.6
Negative/defective - mmol/L	15	11.2	4.7
Negative/negative - mmol/L	12	17.5	3.3

HeFH, heterozygous familial hypercholesterolaemia; HoFH, homozygous familial hypercholesterolaemia; LDL-C, low-density lipoprotein cholesterol.

In total, 496 (17.3%) patients diagnosed with HeFH, either genetically (489 (17.6%)) or clinically (7 (4.5%)), had LDL-C levels of more than twice the upper limit of normal, above 6.7 mmol/l or, if LDL-C was missing, a total cholesterol level of above 8.7 mmol/l. For patients with a negative variant this was present in 29.3% (*n* = 352) whereas in patients with a defective variant this was the case in 9.1% (*n* = 137).

Worrisome for making the diagnosis clinically is that LDL-C levels below 4.0 mmol/l were found in 399 (26.4%) HeFH patients with a defective variant and in 87 (7.2%) HeFH patients with a negative variant. Based on their LDL-C value, these children would not fulfill the clinical criteria for HeFH [13]. Some of them even had normal LDL-C levels, occasionally induced by a combination of a pathogenic and a favorable ‘counter’ mutation, causing hypobetalipoproteinemia, Proprotein convertase subtilisin/kexin type 9 (PCSK9) loss of function or hyperalphalipoproteinemia.

**FIGURE 3:**
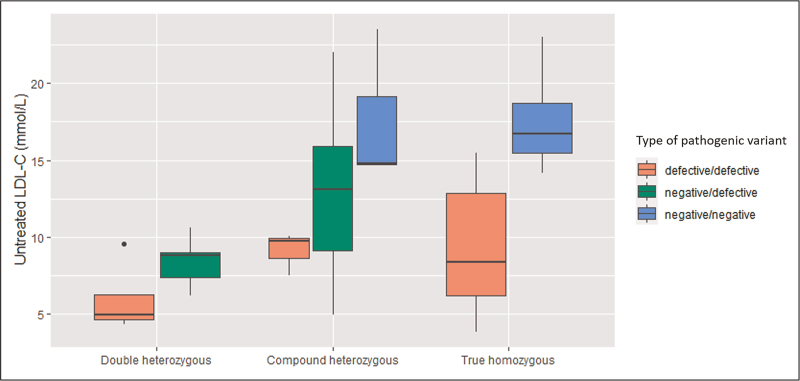
LDL-C levels by type of pathogenic variant and homozygous type.

In total, 41 patients at our center were diagnosed with HoFH of whom 14 patients had two defective variants, 15 patients a defective and a negative variant, and 12 two negative variants (Supplementary Table 2). The median [IQR] LDL-C is 12.0 [7.5-15.5] mmol/L. Mean LDL-C levels are presented in Fig. 3 and Table 3. When grouping the patients traditionally in double heterozygous, compound heterozygous and true homozygous FH, mean LDL-C levels were 7.3 (2.3), 12.9 (5.2) and 13.9 (5.4) mmol/l, respectively, and there is no significant difference between compound heterozygous and true homozygous FH (=1.00). Though grouping the HoFH patients by type of pathogenic variant showed profound mean LDL-C differences of 8.4 (3.6), 11.2 (4.7) and 17.5 (3.3) mmol/l for patients with two defective, patients with one negative and one defective and patients with two negative variants, respectively (Figure 2).

**Table 3:** Mean (SD) LDL-C for HoFH patients (mmol/L)

Mean (SD) LDL-C	Overall	Defective/defective	Negative/defective	Negative/negative
Overall - mmol/L	**12.1 (5.4)** **(n** **=** **41)**	**8.4 (3.6)** **(n** **=** **14)**	**11.2 (4.7)** **(n** **=** **15)**	**17.5 (3.3)** **(n** **=** **12)**
Double heterozygous - mmol/L	**7.3 (2.3)** **(n** **=** **9)**	6.0 (2.4) (n = 4)	8.4 (1.7) (n = 5)	x
Comp. heterozygous - mmol/L	**12.9 (5.2)** **(n** **=** **16)**	9.1 (1.4) (n = 3)	12.6 (5.2) (n = 10)	17.7 (5.1) (n = 3)
True homozygous - mmol/L	**13.9 (5.4)** **(n** **=** **16)**	9.4 (4.4) (n = 7)	x	17.4 (2.9) (n = 9)

HoFH, homozygous familial hypercholesterolaemia; LDL-C, low-density lipoprotein cholesterol; SD, standard deviation.

### Cardiovascular disease in relatives by type of underlying pathogenic variant in heterozygous familial hypercholesterolemia patients

**FIGURE 4:**
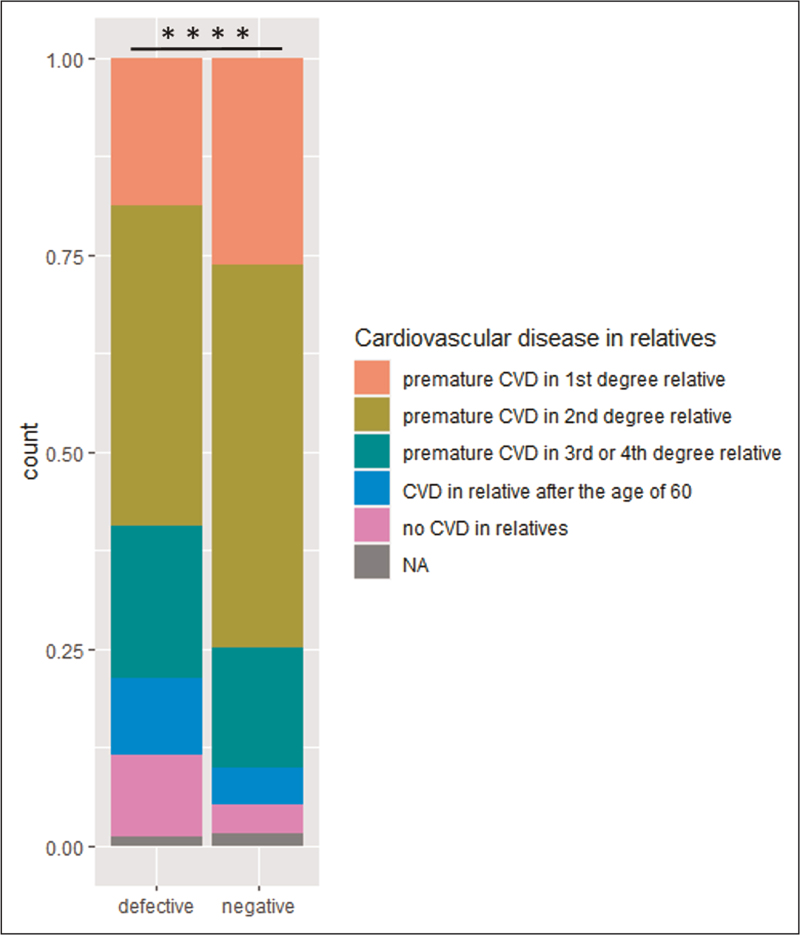
Premature CVD in relatives of patients with genetically diagnosed HeFH.

Figure 4 shows the distribution of CVD in relatives of genetically confirmed HeFH patients at referral, specified for HeFH patients with a negative and those with a defective variant. CVD in relatives was categorized in premature CVD (before the age of 60 years) in respectively first, second, third or fourth degree relatives, CVD after the age of 60 years in relatives and no CVD in relatives. HeFH patients with a negative variant had significant more premature CVD in first and second degree relatives compared to HeFH patients with a defective variant, 75% vs. 59%, *P* = 0.001, whereas no premature CVD (CVD after 60 or no CVD in relatives) is much more frequent in relatives with defective variants than in those with negative variants, 20% versus 8%, *p *=* *<0.001.

## DISCUSSION

Our data show a statistically significant higher mean untreated LDL-C in HeFH patients with a negative variant compared to HeFH patients with a defective variant. More than a quarter of patients with a defective variant did not meet the clinical criteria for FH because their LDL-C level was below 4 mmol/l and the diagnosis of FH would be missed in these children. In addition, more than a sixth of patients with a negative variant had untreated LDL-C levels twice the upper limit of normal and these patients probably will need more intensive lipid lowering treatment than standard to achieve treatment goals.

In HoFH patients, the first thing that strikes us, is that median and mean LDL-C levels of the cohort are below the clinical threshold for HoFH of 13 mmol/l (7); meaning that half of them, clinically expected to be HeFH, turns out to be HoFH when genetic testing is performed. Mean untreated LDL-C levels are the lowest in patients with two defective variants, higher in patients with one defective and one negative variant and the highest in patients with two negative variants. Bringing in type-of-variant make LDL-C levels differ significantly between the groups. The three groups need different treatment strategies and increasing intensity of treatment.

Furthermore, first and second degree relatives of children with HeFH carrying a negative variant showed significant higher presence of premature CVD compared to first and second degree relatives of children with HeFH carrying a defective variant, and significantly lower total absence of premature CVD. This implies the necessity of an earlier and more intensive start of treatment in patients carrying a negative variant.

The differences in mean LDL-C among this large group of HeFH children with negative and defective variants are consistent with what has been described in literature. In a small-scale cohort of 264 children with HeFH, those with negative variants had higher LDL-C levels and a higher prevalence of premature CVD in first-degree relatives [15]. Our group published about 593 children with HeFH with negative, defective and undetermined variants [10] at a time less was known about residual LDLR activity and its implications for treatment, because there were less treatment options than nowadays [16^▪^].

The distribution of HoFH on type of both pathogenic variants in relation to levels of mean LDL-C have not earlier been described in such a large group of children. Our findings are consistent with Kolansky *et al.*[17] who described a group of relatively young HoFH patients, but half of them were adults and many pathogenic variants were classified as undetermined. However, those with negative *LDLR* variants had higher LDL-C levels and an earlier onset of coronary artery disease compared to those with defective variants [17]. Also Stefanutti *et al.*[18] and Sanchez-Hernandez *et al.*[19] showed that premature CVD was more present at an earlier age in HoFH patients with negative variants compared to those with defective variants.

Screening in children for FH will now be widely introduced in Europe, since the European Commission Public Health Best Practice Portal recognized FH pediatric screening as one on the best practices in noncommunicable disease prevention. Different types of screening strategies will be used and hopefully lead to tailored treatment [20,21^▪^]. According to Peretti *et al.*, genetic testing for HeFH during childhood is preferable over clinical diagnosing, because a genetic diagnosis is better associated with statin treatment in HeFH children, as is a history of CVD in a parent. They state that genetic diagnosis may be useful for CVD prevention in children [22]. The results of our present large cohort study show that genetic testing for FH provides information that may have an impact on clinical practice. Firstly, genetic testing can identify FH patients with modestly elevated LDL-C levels, who would be missed if only clinical criteria are used. Missing out an opportunity to early diagnose these patients could result in no further monitoring and leaving them untreated while LDL-C levels may rise later in life. Secondly, these results show that the type of underlying pathogenic variant is not only a reliable predictor for LDL-C levels in HeFH and HoFH patients, but also for the prediction of premature CVD in their families. This emphasizes the importance of early and appropriate lipid-lowering treatment to decrease the risk of premature CVD, specifically in children with negative variants. Thirdly, the type of underlying variant can predict the efficacy of treatments. For HoFH patients with two negative variants, lipid-lowering drugs targeting the LDLR will hardly be effective. For these patients, nowadays treatments working independently of the LDLR pathway are effective [23,24]. Finally, the group with clinically diagnosed HeFH without a known pathogenic variant has even lower mean LDL-C levels compared to the group with a genetically confirmed defective variant. Factors other than the disturbance of the LDLR function might be causative in this group, and need further investigation. Recently it was demonstrated that functional variants in genes regulation the triglyceride metabolism can produce an FH-like phenotype in combination with somewhat lower LDL-cholesterol levels [25^▪^].

## CONCLUSION

This study further substantiated the genotype–phenotype correlation regarding LDL-C levels and CVD in relatives in this large pediatric population. On the one hand, this study confirmed the importance of genetic testing for FH and its impact on the clinical management of the disorder, but on the other hand also demonstrated the need for further in-depth research of the genetics and lipoprotein metabolism to improve the entire disease management of FH.

## Acknowledgements

*None.*

*Author contributions: M.D.R. and A.W. performed the conceptualization. M.D.R. wrote the manuscript. J.C.D. and A.W. provided the data and supervised the writing of the manuscript. All gave final approval and agreed to be accountable for all aspects of work ensuring integrity and accuracy.*

### Financial support and sponsorship

*The authors received no financial support for the research, authorship, and/or publication of this article.*

### Conflicts of interest

*A.W. reports funding from Amryt for participation on a Data and Safety Monitoring Board and for research from Amgen, Regeneron, Novartis, and Silence Therapeutics. M.D.R. and J.C.D. declare no conflicts of interests.*

## Supplementary Material

Supplemental Digital Content

## REFERENCES AND RECOMMENDED READING

Papers of particular interest, published within the annual period of review, have been highlighted as:

▪ of special interest
▪▪ of outstanding interest
